# Toxins and the Kidneys: A Two-Way Street

**DOI:** 10.3390/toxins17120578

**Published:** 2025-12-01

**Authors:** Louis L. Huang, Anthony Longano, Lawrence P. McMahon

**Affiliations:** 1 Department of Renal Medicine, Eastern Health, Box Hill, VIC 3128, Australia; lawrence.mcmahon@monash.edu; 2 Department of Medicine, Eastern Health Clinical School, Monash University, Box Hill, VIC 3128, Australia; 3 Department of Anatomical Pathology, Eastern Health, Box Hill, VIC 3128, Australia

**Keywords:** nephrotoxin, acute kidney injury, chronic kidney disease, uraemic toxin

## Abstract

Nephrotoxin-mediated kidney injury is an important clinical problem, as it can lead to acute kidney injury and chronic kidney disease. Both entities are associated with significant morbidity, increased hospitalisation, healthcare utilisation, and cardiovascular mortality. With the loss of kidney function, there is an accumulation of uraemic toxins, of which the protein-bound toxins—indoxyl sulphate and p-cresyl sulphate—can further inflict damage to the kidneys and the cardiovascular system, culminating in a vicious cycle. Therefore, it is imperative that clinicians have a firm understanding of the common causes and mechanisms of toxin-mediated kidney injury, as well as their clinical presentations and histopathologic features, in order to reduce the prevalence of this pernicious condition.

## 1. Introduction

Kidneys are susceptible to injury from a wide array of aetiologies. A convenient clinical framework to conceptualise kidney injury is to ascertain whether the insult is pre-renal (e.g., hypovolaemia, cardiac failure), intrinsic (e.g., glomerulonephritis, interstitial nephritis), or post-renal (e.g., obstruction, often from stones or prostatic hypertrophy). Useful though it has proven to be, one deficiency in such a framework is that it has little relevance to time. Reversible or irreversible changes might occur within days or, alternatively, over years.

Acute kidney injury (AKI), defined as a sudden increase in serum creatinine and reduction in urine output within 7 days [1], is a frequent cause of kidney disease and is associated with increased healthcare utilisation including hospitalisation, longer duration of admissions, and an increased mortality rate [2,3]. Nephrotoxin-mediated AKI contributes to approximately 20–25% of all AKI episodes in hospital [4]. While the causal relationship between nephrotoxin exposure and AKI can be difficult to establish due to the heterogenous nature of the condition, some toxins have been consistently reported to cause kidney injury, with robust mechanistic studies to strengthen the association. Nephrotoxin-mediated AKI is often avoidable and a sound understanding of the potential causes is important. If the AKI is not recognised and the injurious toxin not promptly withdrawn, AKI can progress to irreversible and chronic kidney disease (CKD). One consequence of CKD is the accumulation of uraemic toxins, a group of molecules with varying sizes and biochemical properties that can potentially perpetuate injury to the kidneys, as well as other organs such as the heart and the vasculature [5].

Kidney injury, be it acute or chronic, is strongly and uniquely associated with heightened cardiovascular morbidity and mortality [6,7]. This review describes how nephrotoxins can injure kidneys and how injured kidneys thereby hurt other organs. We summarise the more common causes of nephrotoxin-mediated AKI, the transition from AKI to CKD with the associated accumulation of uraemic toxins, and how uraemic toxins in turn contribute to kidney injury to continue the pernicious cycle that is known as CKD.

## 2. Nephrotoxin-Mediated Acute Kidney Injury

The list of nephrotoxin-mediated AKI is lengthy and can be bewildering, as even a single toxin can cause different types of kidney damage. A more logical framework to understand nephrotoxin-mediated AKI can be achieved by classifying toxin-mediated AKI first as endogenous or exogenous, and second by the location of its the noxious effects on the kidneys (renal tubulo-interstitial, glomerular, or microvascular compartments). It is also important to consider other causes of kidney injury, e.g., from hypovolaemia or uncontrolled hypertension, that can occur alongside toxin-induced AKI.

### 2.1. Exogenous Nephrotoxins Affecting Renal Tubulo-Interstitial Compartment

**Figure 1:**
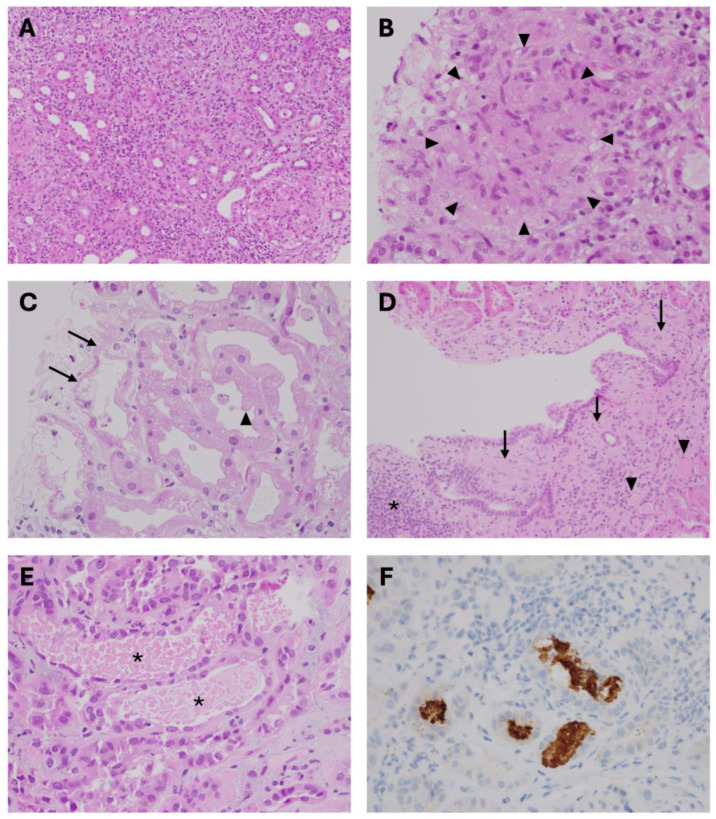
(**A**) Nephrotoxin-mediated acute interstitial nephritis with a dense mononuclear infiltrate and rare eosinophils in the tubulo-interstitial compartment of the renal cortex (haematoxylin and eosin [H&E] stain, original magnification ×100). (**B**) Granuloma formation within the interstitium (within arrowheads) after chronic exposure to nephrotoxin (H&E stain, original magnification ×400). (**C**) Acute tubular injury, with flattening of the tubular epithelium (arrows), bleb formation (arrowhead), and cellular debris within the tubular lumen (H&E stain, original magnification ×400). (**D**) Chronic lithium-induced nephropathy, resulting in interstitial fibrosis (arrows), tubular atrophy (arrowheads), and microcystic change, along with commensurate lymphocytic interstitial inflammation (asterisk) (H&E stain, original magnification ×200). (**E**) Acute tubular injury with intraluminal myoglobin cast formation (asterisks), flattening of the tubular epithelium due to rhabdomyolysis (H&E stain, original magnification ×400). (**F**) Immunohistochemistry stain for myoglobin, with haematoxylin counterstain, demonstrating myoglobin casts obstructing the tubular lumen (myoglobin stain, original magnification ×400).

This category contains different toxins, some of which are prescription medicines, while others may be available over the counter (Table 1). The common downstream effect of these toxins is interstitial inflammation, with or without tubular cell damage. Perhaps the most prevalent example is the use of beta-lactam antibiotics such as penicillin, causing acute interstitial nephritis [8,9]. Other antimicrobial medications that can cause acute interstitial nephritis include cephalosporins, rifampicin [10], quinolones including ciprofloxacin [11] and sulphonamides such as trimethoprim–sulfamethoxazole [12]. Other classes of medications that cause interstitial nephritis include non-steroidal anti-inflammatory drugs (NSAIDs) including ibuprofen and cyclo-oxygenase-2 inhibitors [13,14]; proton pump inhibitors such as omeprazole [15]; anti-neoplastic agents such as immune checkpoint inhibitors [16]; herbal remedies including *Aristolochia* species [17]; and diuretics, including furosemide [18]. Pathologically, acute interstitial nephritis is an allergic-type reaction that results in inflammation of the interstitial compartment of the kidneys (Figure 1A). The acute nature of the process is indicated by the presence of interstitial oedema, which appears as a blue-grey discolouration under light microscopy on haematoxylin and eosin staining. Typically, the inflammatory infiltrate comprises lymphocytes and monocytes, with scattered eosinophils, and can range from mild to severe in its intensity or breadth of coverage. Granulomas can be present (Figure 1B), while tubular epithelial cell damage may also be observed [19]. Clinically, the affected person may have no revealing features and the classical triad of fever, rash, and eosinophilia, often touted in medical textbooks, is much less commonly observed in clinical practice [20]. Urine analysis often demonstrates white cell casts and should be free of erythrocytes or protein.

Nephrotoxins can also mediate a predominantly tubular cell injury that frequently causes AKI. The main histopathological findings of acute tubular injury include flattening of the tubular epithelial cells, loss of the brush border, bleb formation of the apical cytoplasm, and filling of the tubular lumen with cellular debris (Figure 1C). If the offending toxin is not promptly removed, acute tubular necrosis (ATN) ensues. The aminoglycoside antibiotic, gentamicin, is the most widely recognised cause of direct toxin-mediated ATN [21]. It results in accumulation of filtered gentamicin within the Golgi apparatus, endosomes and endoplasmic reticulum of renal tubular cells, which induces oxidative stress and mitochondrial dysfunction, culminating in activation of cell death pathways [22]. Other toxins that cause direct tubular injury are listed in Table 1 and include glycopeptide antibiotics such as vancomycin [23]; antiviral agents including cidofovir, foscarnet and tenofovir [24,25]; antineoplastic agents such as cisplatin [26]; zoledronic acid [27]; and radiocontrast media [28]. Some or all of these toxins can also affect the kidneys in other ways (vide infra). Radiocontrast agents, for example, also cause prolonged medullary vasoconstriction, an effect aggravated by small vessel disease and volume depletion, resulting in further tubular ischaemic injury [29].

Clinically, patients with renal tubular injury frequently present with electrolyte disturbance. A prototypical example of this is the Fanconi syndrome, where proximal tubular cells that are responsible for reabsorption of fluid and electrolytes are damaged, leading to amino aciduria, glucosuria, phosphaturia, and renal potassium and bicarbonate wasting [30]. Independent of such features, sustained insult to the renal tubular cells can lead to established ATN, which often presents with oliguria, signs of salt and water excess manifesting as hypertension, pitting oedema, an elevated jugular venous pressure, and an increase in serum urea and creatinine [31]. On microscopy, urine analysis demonstrates muddy brown, granular casts, usually without red cells. Fractional excretion of sodium > 2% is more consistent with ATN (compared with <1% in pre-renal causes of AKI), although interpretation is often limited by the presence of pre-existing renal impairment or concurrent diuretic use [31]. With prompt withdrawal of the offending nephrotoxin and optimal supportive care (blood pressure control and achieving an euvolaemic state), recovery of ATN usually follows within two to three weeks due to regeneration of renal tubular epithelial cells. However, recovery can be delayed or, on occasion in older patients, absent—especially when the insult is severe or prolonged. Acute cortical necrosis (ACN) rarely occurs in extreme cases (e.g., profound sepsis and catastrophic post-partum haemorrhage) and is characterised by red urine containing both haemolysed and intact erythrocytes; useful recovery of kidney function in ACN is unusual [32].

Long-term exposure to nephrotoxins that affect the renal tubulo-interstitium can cause interstitial fibrosis. A classic example of this is the treatment of bipolar affective disorder using lithium salts. Lithium is an excellent mood stabiliser and has been used since the mid-20th century; however, it has several adverse effects on the kidneys, including impaired function of the principal cells in the distal nephron, causing nephrogenic diabetes insipidus [33] and CKD through the development of interstitial fibrosis [34]. Case series have described patients on long-term lithium to have reduced kidney function; reduced urine concentration capacity; and histological changes in tubular microcyst formation and interstitial fibrosis on renal biopsy (Figure 1D) [35]. The mechanism of lithium-induced interstitial fibrosis has been informed by animal studies. Rats given oral lithium for 6 months developed renal interstitial inflammation (predominantly macrophages) and myofibroblast infiltration, with upregulation of pro-fibrotic mediators such as transforming growth factor-β (TGF-β), connective tissue growth factor [36], and platelet-derived growth factor-β, resulting in excessive collagen deposition, culminating in interstitial fibrosis and kidney dysfunction [37]. From a clinician’s perspective, patients treated with lithium salts for more than a decade are more likely to have progressive kidney complications [38]. Nephrogenic diabetes insipidus presents with polyuria > 4 L per day (on occasion >15 L daily), polydipsia and dilute urine with urine osmolality generally <300 mOsm/kg. Treatment with amiloride, an inhibitor of epithelial sodium channels in the distal nephron, has been shown to reduce polyuria in patients on lithium, as well as ameliorating renal fibrosis in animal models [37]. The decision to stop lithium due to its nephrotoxicity requires judicious deliberation and individualised care, as the adverse effects of the underlying mood disorder might justify drug continuation.

### 2.2. Endogenous Nephrotoxins Affecting Renal Tubular Cells

Proteins that are intrinsic to the human body can also cause kidney injury. Examples are listed in Table 1 and include myoglobin, haemoglobin, bile salts, uric acid, and phosphate [39,40,41,42]. These proteins are predominantly intracellular; however, in the setting of a sudden and massive increase in the concentration of these proteins (e.g., with rhabdomyolysis or haemolysis), renal tubular epithelial cell damage occurs and can manifest as electrolyte disturbance or AKI.

The release of myoglobin from a damaged muscular sarcolemma sheath is a prominent cause of AKI. In Western societies, it is most often due to prolonged recumbency from illicit drugs, or a crush injury due to earthquake or explosion, and is clinically coined rhabdomyolysis [43]. Historically, such kidney injury was well described during wartime, extending back to the Napoleonic encounters in the early 19th century. Non-traumatic causes of rhabdomyolysis are also well known and include prolonged exercise especially in the heat [44], use of illicit drugs such as cocaine [45], statin therapy [46], inflammatory myopathies, severe hypokalaemia, and post-infectious aetiologies [47]. Approximately 10–40% of patients presenting with rhabdomyolysis develop AKI [48], often with risk factors such as volume depletion and CKD. Once myoglobin is released into the circulation, it is freely filtered by the glomerulus and dissociates into ferrihaemate and globin in the urinary space [49]; the former causes direct tubular cell toxicity by the abundance of free ferrous (Fe^2+^) iron and subsequent formation of reactive oxygen species [39], while the latter binds uromodulin proteins and results in tubular cast obstruction. Histologically, acute tubular injury, including tubular necrosis and tubular casts, can be seen with haematoxylin and eosin staining, as described earlier (Figure 1E). The pathognomonic feature of rhabdomyolysis is found on positive immunohistochemistry staining for myoglobin within the tubular casts (Figure 1F). Clinically, in Western nations, patients with rhabdomyolysis and AKI usually present with a history of a forced (crush injury) or drug-induced prolonged immobilisation, maroon-coloured urine, muscle weakness and oliguria [47]. Urine analysis is positive for ‘blood’ (haem) on dipstick but lacking erythrocytes under microscopy, and pigmented granular casts. Additional laboratory features include significantly elevated creatine kinase, usually between fifty and a hundred thousand IU/L, hyperkalaemia, hypocalcaemia, and elevated liver enzymes. A rapid and sustained rise in serum urea and potassium concentrations can demand daily haemodialysis in severe cases. Initial and profound hypocalcaemia can be associated with intracellular sequestration in the sarcoplasmic reticulum and hyperparathyroidism which, after one to two weeks, often evolves into protracted hypercalcaemia, which is relatively resistant to standard measures [50].

Massive intra-vascular haemolysis can also induce haem-pigment mediated AKI similar to that of myoglobin, where the proximal tubular cells are injured by reactive oxygen species stemming from the disturbance in the redox state due to Fe^2+^ iron overload [40]. Causes of haemolysis resulting in haemoglobinuria include autoimmune haemolytic anaemia; paroxysmal nocturnal haemoglobinuria [51]; thalassaemia [52]; infections such as malaria [53]; medications such as cephalosporin antibiotics [54]; non-steroidal anti-inflammatory drugs such as diclofenac [55]; immune checkpoint inhibitors [56]; and dapsone [57], especially in the setting of glucose-6-phosphate dehydrogenase deficiency [58].

Another set of endogenous molecules that can cause kidney injury when concentrations are suddenly and significantly elevated are uric acid and phosphate [42,59]. The clinical scenario where both molecules are increased markedly is the tumour lysis syndrome. Typically, this syndrome occurs during induction treatment of bulky, chemotherapy-sensitive haematological cancers such as Burkitt’s lymphoma or diffuse large B-cell lymphoma [59,60]. As cancer cells are destroyed, their intracellular contents, including phosphate and nucleic acids, which are catabolised to uric acid by xanthine oxidase, are released into the circulation in extremely high concentrations. Prior to the routine use of xanthine oxidase inhibitors as preconditioning therapy, uric acid crystals would precipitate in the acidic urine due to its poor solubility, resulting in deposition within the renal tubules. What followed was a combination of renal tubular occlusion from uric acid crystals, as well as a pro-inflammatory and pro-apoptotic response—involving upregulation of cytokines such as intracellular adhesion molecule-1 and caspase-1—that resulted in further tubular cell injury and apoptosis [61].

As the current practice of pre-conditioning with allopurinol or rasburicase and volume repletion with intravenous fluids has become standard of care, AKI from tumour lysis syndrome is now more likely due to the nephrotoxic effect of hyperphosphataemia. Concentrated phosphate levels can precipitate with calcium, resulting in calcium phosphate crystal deposition, acute tubular injury, and luminal obstruction. Cell culture and animal models of hyperphosphataemia have demonstrated tubular epithelial cell flattening, vacuolisation, loss of cell polarity, as well as enhanced macrophage infiltration of the renal interstitium via upregulation of monocyte chemoattractant protein-1 [62]. Acute phosphate nephropathy can also develop from an exogenous source of phosphate in high concentrations. This can be observed in patients using phosphate-containing bowel preparations prior to colonoscopy [42]. Findings on renal histopathology typically include tubular atrophy, acute tubular necrosis, and deposition of calcium phosphate crystals, best seen with von Kossa staining.

There is an association between bile salt excess and AKI, termed cholemic nephropathy or bile cast nephropathy [63]. In patients with advanced chronic liver disease or obstructive cholestasis, the physiologic secretion of bile salts into the duodenum is impeded, leading to significantly elevated serum levels. In this setting, the kidneys assume principal responsibility for the excretion of bile salts, including increased tubular secretion. However, bile salts are directly toxic to tubular cells, with mechanisms akin to uric acid and phosphate nephropathy, as well as tubular obstruction from pigmented casts [41]. The most striking pathologic feature of cholemic nephropathy is that of yellow discolouration of the kidneys, which turns green after formalin fixation. Microscopically, pigmented casts (yellow/green) are observed, along with varying degrees of acute tubular injury. Clinically, patients are jaundiced and may have a history of pale stools, dark urine, and liver disease. Treatment is mainly directed at overcoming the obstructive jaundice to reduce circulating levels of bile salts, as well as supportive management of the AKI such as volume repletion and cessation of other nephrotoxic medications [64].

### 2.3. Nephrotoxins Affecting Glomeruli and Renal Microvasculature

Toxins that affect the glomeruli and microvasculature are less common than those causing tubular injury. Both renal compartments are exposed to the same nephrotoxin; however, there are differences in toxin concentration due to water reabsorption and reduced oxygen tension along the renal tubules, thus favouring glomerular survival. Nevertheless, the glomeruli are still targets for nephrotoxicity, with the predominant clinical manifestations being the nephrotic syndrome and thrombotic microangiopathy.

**Figure 2:**
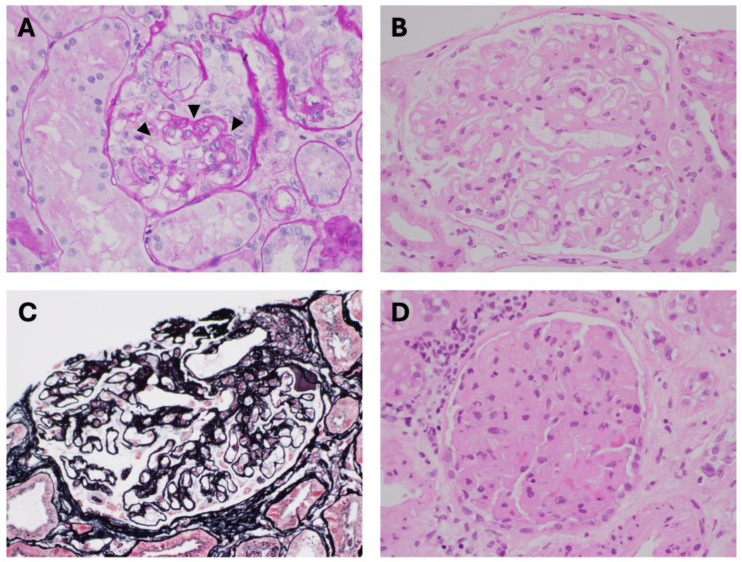
(**A**) Collapsing focal and segmental glomerulosclerosis, showing shrunken glomerular tuft obliterating capillary loops (arrowheads) (periodic acid-Schiff stain, original magnification ×400). (**B**) Thickened glomerular capillary loops due to membranous nephropathy (H&E stain, original magnification ×400). (**C**) Membranous nephropathy with thickened glomerular capillary loops, including spike formation arising from the epithelial side of the glomerular basement membrane (silver methenamine stain with Masson trichrome counter stain, original magnification ×400). (**D**) A ‘bloodless’ glomerulus, featuring total obliteration of capillary loops due to endothelial swelling, as well as fibrin microthrombi (H&E stain, original magnification ×400).

The nephrotic syndrome is characterised by proteinuria > 3.5 g/day, hypoalbuminaemia, oedema, and hypercholesterolaemia. The main primary (intrarenal) conditions responsible for nephrotic syndrome include focal and segmental glomerulosclerosis (FSGS), membranous nephropathy, and minimal change disease. Toxins can mimic each of these primary kidney conditions and are shown in Table 2. Bisphosphonates such as pamidronate and zoledronic acid, interferon, and heroin can all cause FSGS [65,66,67,68]. Due to its inhibitory effects on bone resorption by osteoblasts, intravenous pamidronate is often used to treat severe hypercalcaemia associated with cancers or high-turnover bone disease, such as Paget’s disease. With pamidronate, the incidence of FSGS is low; however, when administered at high concentrations and repeatedly, the condition can develop over 4 to 40 months [69,70]. While the mechanism is unclear, the prevailing hypothesis is that of podocyte cell apoptosis, as an off-target effect aimed at osteoclasts [71]. Kidney biopsies arising from case reports have demonstrated a variety of histologic changes, including FSGS where select glomeruli have sections of sclerosis and collagen deposition, collapsing FSGS with segmental collapse of the glomerular tuft (Figure 2A), as well as minimal change disease [72]. Areas of glomerular sclerosis are best observed under Jones methenamine silver staining or periodic acid-Schiff staining. Electron microscopy usually demonstrates podocyte foot process effacement of <80%, reflecting the secondary nature of the toxin-induced podocytopathy. Other effects of bisphosphonates, particularly zoledronic acid, is tubular toxicity, leading to acute tubular necrosis and AKI [71].

Another histologic pattern of toxin-induced glomerular injury causing the nephrotic syndrome is membranous nephropathy. This association has been recognised since the 1970s, in the setting of chronic gold salts treatment for rheumatoid arthritis [73]. Other medications implicated include NSAIDs [74], penicillamine [75], and mercury from topical skin-lightening ointments [76]. Primary membranous nephropathy is usually an antigen-antibody mediated disease, caused by anti-phospholipase A2 receptor antibodies targeting the podocytes [77]. However, these are usually absent in secondary, nephrotoxin-induced membranous nephropathy. By employing laser capture microdissection of glomeruli, newer antigens have been identified in nephrotoxin-induced membranous nephropathy and include neural epidermal growth factor-like 1 (NELL1) and proprotein convertase subtilisin/kexin type 6 (PCSK6) [78]. For example, chronic exposure to NSAIDs has been associated with secondary membranous nephropathy, which can present as the nephrotic syndrome associated with the PCSK6 antigen, while penicillamine and mercury exposure have been linked to antibodies against NELL1 [78]. Histologically, the classic findings are thickened glomerular capillary loops (Figure 2B), with subepithelial deposits that are best seen on silver methenamine stain (Figure 2C) under oil emersion. Immunofluorescence usually demonstrates diffuse granular staining for IgG along the basement membrane of glomerular capillaries. In contrast to primary membranous nephropathy where IgG4 staining predominates, IgG1 is usually the dominant subtype in nephrotoxin-induced membranous nephropathy [74]. Examination under electron microscopy commonly shows incomplete podocyte foot process effacement and electron dense deposits in the subepithelial aspect of the glomerular basement membrane. Withdrawal of the offending nephrotoxin often leads to remission of the nephrotic syndrome, although on occasion, treatment with corticosteroids may be required for complete remission.

Minimal change disease, as the name infers, is a cause of the nephrotic syndrome, characterised by the normal appearance of glomeruli under light microscopy. When examined under electron microscopy, pathognomonic findings of podocyte foot process effacement can be seen—albeit incomplete—due to the secondary nature of minimal change disease attributable to nephrotoxins. Common causes of toxin-induced minimal change disease are listed in Table 2, including exposure to NSAIDs, beta-lactam antibiotics such as penicillins and cephalosporins, rifampicin, lithium, as well as pamidronate [69,79,80,81].

Thrombotic microangiopathy (TMA) is a distinct pathologic process that affects small blood vessels, including the glomeruli and renal microvasculature. The pathognomonic features of TMA include endothelial injury, platelet aggregation, and microthrombus formation causing vessel luminal obstruction, followed by microangiopathic haemolytic anaemia and distal organ ischaemia [82]. There is usually an elevated serum creatinine, thrombocytopaenia, normocytic anaemia, evidence of haemolysis such as absent circulating haptoglobin, fragments and schistocytes on peripheral blood smear, elevated lactate dehydrogenase, and proteinuria without haematuria. Levels of ADAMTS-13 are usually normal in nephrotoxin-induced TMA. Although nephrotoxin-induced TMA only contributes to a small proportion of cases of TMA, it is usually readily identifiable on history taking and review of medications (Table 3), with culprits including quinine [83], calcineurin inhibitors [84], vascular endothelial growth factor inhibitors such as bevacizumab [85], and cocaine [86]. Excessive use of quinine for muscle cramps or intake of tonic water can lead to TMA due to the development of platelet autoantibodies [82]. Calcineurin inhibitors, such as tacrolimus and cyclosporin, are ubiquitously used in organ transplantation, as well as steroid-sparing agents to treat autoimmune conditions; however, their use has also been associated with a dose-dependent renal-limited TMA [84]. Under the light microscope, renal TMA comprises a bloodless appearance to the glomeruli due to swelling of the endothelial cells and fibrin thrombus formation within the capillary loops (Figure 2D). Fibrin and fibrinogen can also be readily identified under immunofluorescence microscopy, affecting both glomeruli and arterioles. Without withdrawal of the offending agent, chronic changes of renal TMA can manifest, including tram-tracking of the glomerular basement membrane and arteriolar myointimal hyperplasia [87].

## 3. AKI to CKD Transition and Accumulation of Uraemic Toxins

**Figure 3:**
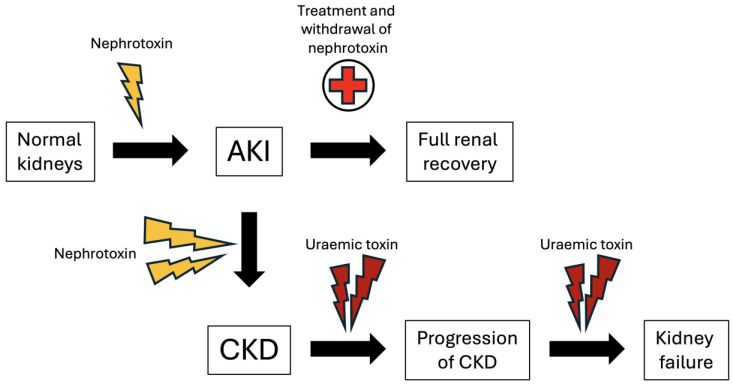
Nephrotoxins cause an initial acute kidney injury that can resolve if identified early and treated promptly with withdrawal of the offending nephrotoxin. However, repeated or sustained exposure to nephrotoxins results in the progression to chronic kidney disease, with reduction in renal clearance of uraemic solutes. Protein-bound uraemic nephrotoxins such as indoxyl sulphate and p-cresyl sulphate accumulate and promote progressive kidney damage, culminating in kidney failure.

There is an expectation that a mild case of AKI conferred by nephrotoxins is followed by a complete recovery of kidney function (Figure 3). However, evidence increasingly suggests this is not the case. Acute kidney injury is consistently associated with increased mortality, longer hospital admissions, and progression to CKD [1]. In a prospective study of AKI with long-term follow-up, patients with AKI from any cause were up to four times more likely to develop incident CKD [6]. The converse is also true, where patients with CKD are also at higher risk of AKI and heightened mortality risk [5]. Exact mechanisms of the AKI to CKD transition remain to be elucidated; however, several risk factors and pathologic mechanisms have been hypothesised. A prevailing theory of AKI to CKD progression is an imbalance of meaningful cellular repair versus maladaptive repair, causing cell death and fibrosis of the kidney [88]. Rodent models of nephrotoxic AKI using cisplatin, high-dose folic acid, and aristolochic acid have provided important insights into the pathogenesis of AKI to CKD transition [89,90,91]. These studies posit that the kidney recovers from a short-lived toxic insult, as inflammation subsides and tubular epithelial cells regenerate [92]. However, in the setting of overwhelming or persistent nephrotoxin exposure, pathological processes including cell cycle arrest, mitochondrial dysfunction, activation of the renin angiotensin–aldosterone axis, and apoptosis/necroptosis occur, resulting in the recruitment of an inflammatory infiltrate comprising neutrophils, macrophages, and lymphocytes [92]. This culminates in a pro-fibrotic environment, where polarised macrophages of the M2 phenotype secrete cytokines such as TGF-β and epidermal growth factor, leading to the conscription of activated myofibroblasts from resident fibroblasts and neighbouring pericytes [93,94]. These cells deposit excessive amounts of extracellular matrix proteins such as collagen within the kidney interstitium and glomeruli, resulting in irreversible renal sclerosis and loss of kidney function [93]. Clinically, recovery from nephrotoxin-induced AKI is dependent on the severity and duration of the initial insult, as well as the frequency AKI recurrence. Other risk factors include older age, presence of pre-existing CKD from diabetic nephropathy or hypertension that may coexist with AKI (kidney reserve), proteinuria [95], genetic and epigenetic factors [96,97].

As CKD develops, there is an accumulation of uraemic toxins due to reduced excretion through impaired glomerular filtration and tubular secretion (Figure 3). Currently, according to the European Uremic Toxin Work Group, approximately 90 uraemic toxins have been identified [98], based on a principle that extends from Koch’s postulates for the validation of pathogenic organisms [99]. Uraemic toxins are required to be chemically quantifiable, have increased serum levels during diseased states, display harmful biological effects, and result in restoration of health when adequately removed. Broadly speaking, uraemic toxins can be classified by their physicochemical properties, which include water-soluble, low-molecular-weight toxins < 500 Da, such as urea and creatinine; middle molecules 500–12,000 Da such as β_2_ microglobulin and fibroblast growth factor-23; and protein-bound uraemic toxins such as p-cresyl sulphate and indoxyl sulphate [98].

## 4. Uraemic Toxins Affecting Kidneys and Beyond

The accrual of uraemic toxins leads to what is known as the uraemic syndrome, a systemic illness manifesting clinically as lethargy, weakness, pruritus, anorexia, nausea, and vomiting. When left untreated, severe complications such as pericarditis, neuropathy, or death can occur. The exact uraemic toxin or toxins that cause the uraemic syndrome have not been identified, although it is likely due to the combined action of several compounds rather than a singular agent. It remains a scientific and medical paradox that despite much research, the two water-soluble molecules ubiquitously and historically used globally to ascertain kidney dysfunction, namely urea and creatinine, have not been confirmed as uraemic toxins according to the previously mentioned Koch’s postulates (vide supra). Creatinine is a molecule with a molecular weight of 113 Da that is produced by the hepatic metabolism of creatine from muscle. High levels of creatinine have been shown to impede chloride channels that negatively affects myocardial contractility [100], but studies have not demonstrated direct nephrotoxicity. Urea, a 60 Da water-soluble molecule, was considered biologically inert for several decades after its discovery. However, laboratory research in the past two decades has shown urea to induce oxidative stress and pro-inflammatory molecule, protein kinase C, in renal medullary cells [101,102], as well as promoting insulin resistance through upregulation of reactive oxygen species and adipokines [103]. Despite these suggestive findings in animal models and cell culture studies, the role of urea in the uraemic syndrome and direct kidney injury has not been forthcoming in clinical research. Administration of additional urea to haemodialysis patients was not associated with meaningful changes to their symptomatology [104], while enhancing urea clearance by increasing the dialysis prescription was not associated with reduced patient mortality, residual kidney function, or quality of life [105,106]. Due to a lack of robust, causative evidence linking water-soluble uraemic toxins with kidney or patient outcomes, the focus has shifted to other uraemic toxins [107].

The archetypical example of a middle molecule uraemic toxin is β_2_ microglobulin, with a molecular weight of approximately 12,000 Da. Retention of β_2_ microglobulin in CKD causes macro-deposition of misfolded fibrils resulting in dialysis-related amyloidosis. This can manifest as carpal tunnel syndrome, arthropathy (particularly shoulders and neck), and associations with increased cardiovascular mortality [108]. However, there is no direct evidence linking β_2_ microglobulin and kidney toxicity.

In contrast, protein-bound uraemic toxins (PBTs) including indoxyl sulphate (213 Da) and p-cresyl sulphate (188 Da) have been demonstrated to cause direct renal toxicity. These toxins are formed through the metabolism of tryptophane, tyrosine, and phenylalanine by the colonic microbiota, and are usually removed by the kidneys through tubular secretion (rather than glomerular filtration) due to tight binding to albumin [109]. As concentrations of PBTs rise, pathological processes that were involved in AKI, as well as the AKI to CKD transition, are seen again, including increased oxidative stress, inflammation, and fibrosis [110,111]. In a model of remnant kidney disease, rats with a subtotal nephrectomy were treated with indoxyl sulphate. Concentrations of serum creatinine, urea, and degree of glomerulosclerosis were higher compared with controls [110]. Mechanistic studies demonstrated upregulation of pro-fibrotic gene transcription such as TGF-β, tissue inhibitor of metalloproteinases-1 and collagen within the kidney, as well as in cultured proximal tubular cells, after indoxyl sulphate treatment [111]. There was also an increase in the gene expression for intercellular adhesion molecule-1, a protein that attracts monocytes and macrophages, causing renal inflammation and fibrosis. Another pathway involved in uraemic toxin-mediated kidney injury is through the nuclear factor-κB (NF-κB) pathways. Cultured human proximal tubular cells treated with indoxyl sulphate had increased expression of p53, NF-κB, TGF-β, and Smad3, which induced cellular senescence, reduced tubular cell proliferation, and promoted fibrosis [112,113], while a 5/6 nephrectomy model of CKD in rats treated with a resin binder of PBT reduced the levels of indoxyl sulphate and attenuated NF-κB, p53, and TGF-β expression [112]. Induction of oxidative stress by reactive oxygen species production has also been identified as a pathologic sequelum of PBT accumulation in the kidneys. Both indoxyl sulphate and p-cresyl sulphate have been shown to increase nicotinamide adenine dinucleotide phosphate-oxidase activity, leading to higher levels of H_2_O_2_ and pro-fibrotic signals in human tubular epithelial cell culture studies, with results corroborated in the rat remnant kidney model of CKD [114]. Finally, PBTs have been shown to increase phenotypic change of renal tubular epithelial cells towards a collagen secreting myofibroblast through epithelial to mesenchymal transition (EMT). Both indoxyl sulphate and p-cresyl sulphate can induce EMT through increased expression of Snail, α-smooth muscle actin, combined with down regulation of zonula occluden-1 and E-cadherin, through p38 mitogen-activated protein kinase activity and renin-angiotensin-aldosterone pathways [115,116].

**Figure 4:**
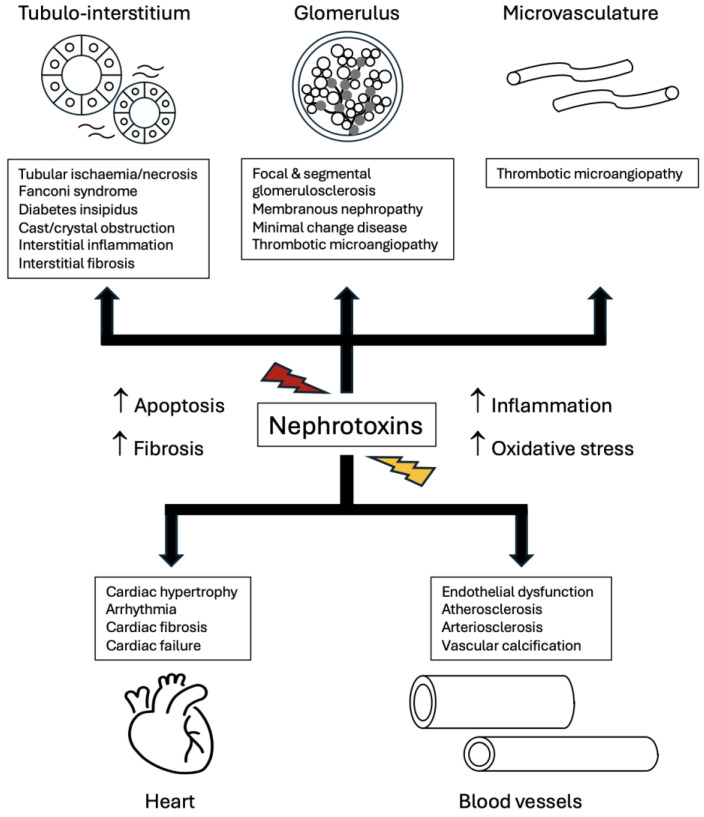
Summary of nephrotoxin-mediated kidney and cardiovascular injury, outlining key pathological processes such as increased oxidative stress, inflammation, apoptosis, and fibrosis, culminating in damage to the different compartments of the kidney, heart, and blood vessels.

Clinically, higher serum levels of PBT have been associated with progression of CKD. In a prospective observational study of CKD patients with a 2-year follow-up, multivariable regression analysis showed that both p-cresyl sulphate and indoxyl sulphate levels were associated with kidney disease progression, defined as a 50% reduction in eGFR or requiring dialysis treatment, after adjusting for confounders such as age, gender, comorbid diabetes, baseline kidney function, and C-reactive protein levels [117]. P-cresyl sulphate, but not indoxyl sulphate, was also associated with all-cause mortality. The association between p-cresyl sulphate and progression of CKD was also observed in another prospective study [118]. In summary, these experimental and clinical studies have provided insights to the deleterious effects of PBTs on the kidneys, causing renal inflammation, fibrosis, and propagating CKD (Figure 4).

There are also extra-renal effects of PBTs, including associations with increased cardiovascular morbidity and mortality [119,120]. This risk association remained, even after adjustment for the traditional Framingham risk factors [120]. Through similar pathological processes that affect the kidneys, namely increased oxidative stress (elevated oxygen free radical activity and reduced nitric oxide bioavailability), pro-inflammatory and fibrotic pathways including NF-κB and TGF-β, respectively, PBTs can cause endothelial dysfunction, promote atherosclerosis, arteriosclerosis, cardiac fibrotic remodelling with increased risk of arrhythmias and cardiac failure [109,121,122]. Human endothelial cell culture experiments have shown that incubation with indoxyl sulphate significantly increased the expression of intercellular adhesion molecule-1 and monocyte chemotactic protein-1 [123], which are key factors involved in the recruitment of macrophages that cause endothelial activation and atheromatous change within blood vessels [124]. Moreover, both p-cresyl sulphate and indoxyl sulphate have been shown to increase the proliferation of vascular smooth muscle cells (VSMCs) via activation of p38 mitogen-activated protein kinase pathways and the platelet-derived growth factor receptor that further contribute towards atherosclerosis [125,126]. In contrast, p-cresyl sulphate impeded the proliferation of cultured human endothelial cells in a dose-dependent manner, resulting in impaired endothelial repair after injury [127]. Another mechanism by which PBTs cause vascular injury is through vascular calcification. Indoxyl sulphate has been shown to induce aortic calcification in a rat model of hypertension [128], as well as promoting the trans-differentiation of cultured human VSMC from a contractile cell to an osteoblastic phenotype that expressed osteogenic proteins such as osteopontin and alkaline phosphatase [129]. Lastly, indoxyl sulphate has been demonstrated to increase both cardiomyocyte hypertrophy and cardiac fibroblast expression of collagen [130], which may contribute to cardiac failure. Taken together, these clinical observations and experimental findings provide robust evidence that PBTs contribute to cardiovascular disease and might be the missing link between kidney disease and heightened cardiovascular mortality (Figure 4).

Despite the growing body of evidence demonstrating the link between PBT and kidney disease progression and cardiovascular disease, there is a paucity of treatments capable of removing PBTs. Their small molecular weight would normally infer a high efficiency of removal through dialysis; however, due to their high protein-binding characteristics, removal by both haemodialysis and peritoneal dialysis are regrettably inadequate. In comparison to urea clearance, PBT clearance is approximately halved in haemodialysis and is one-tenth in peritoneal dialysis [131,132]. Attempts at reducing PBTs by reducing their generation and elimination have also been investigated. Adsorption of PBT in the intestine by AST-120, an oral carbon agent, has been utilised in CKD patients to reduce serum levels of indoxyl sulphate and p-cresyl sulphate, and it is associated with slower progression of CKD and reduced kidney fibrosis [133]. Notwithstanding the early, favourable results, a subsequent, but small randomised clinical trial did not demonstrate a reduction of serum indoxyl sulphate or changes to eGFR and proteinuria with AST-120 treatment over 3 years compared to controls [134]. Displacement of PBTs from albumin is another strategy deployed to improve their removal during dialysis. In a small crossover study involving 17 anuric haemodialysis patients, treatment with loop diuretics increased dialytic clearance of indoxyl sulphate approximately two-fold, while p-cresyl sulphate clearance was increased by approximately three-fold [135]. Modulation of PBT generation from the colon is another possible pathway towards PBT reduction. An observational study of haemodialysis patients demonstrated a marked reduction in p-cresyl sulphate concentration (68-fold) and indoxyl sulphate (35-fold) in those with a history of colectomy compared to patients with intact colons [136]. The authors do not propose performing colectomies to reduce PBT; however, the study does highlight the role of the large intestine and indeed its microbiota in the generation of PBTs. Finally, there is a potential role for dietary fibre to favourably influence the colonic microbiome and reduce the production of PBT in dialysis patients [137], although results thus far have been inconsistent [138]. Evidently, more work is needed to reduce PBTs or ameliorate their downstream pathologic impact to assess the potential benefit on CKD progression. It is also unclear whether adequate removal of circulating PBTs would reduce the elevated clinical risk of morbidity and mortality in uraemic patients.

## 5. Conclusions

This review summarised the effects of the more common nephrotoxins that can result in kidney injury, the complex pathophysiology of the progression from AKI to CKD, and the subsequent accumulation of differing classes of uraemic toxins in CKD, some of which can further aggravate kidney injury and culminate in a perpetuating cycle of kidney damage. To avoid or minimise injury, ascertain nephrotoxin-induced AKI, and reduce the likelihood of progression towards advanced CKD, it is important that clinicians are familiar with the identity and effects of common nephrotoxins. Additionally, better understanding the mechanisms of kidney injury by protein-bound toxins will likely create therapeutic options to break the cycle of ‘kidneys hurt, kidneys hurt’.

## Figures and Tables

**Table 1:** Common nephrotoxins affecting the renal tubulo-interstitial compartment.

Tubulo-Interstitial Disease	Nephrotoxin
Acute interstitial nephritis	Antibiotics, e.g., penicillin, cephalosporins, gentamicin, ciprofloxacin
	Non-steroidal anti-inflammatory drugs
	Proton pump inhibitors, e.g., omeprazole
	Lithium
	Immune checkpoint inhibitors, e.g., pembrolizumab
	*Aristolochia* species
	Furosemide
Acute tubular injury/necrosis	**Exogenous**
	Non-steroidal anti-inflammatory drugs
	Antibiotics, e.g., gentamicin, vancomycin
	Antivirals, e.g., acyclovir, tenofovir
	Antifungals, e.g., amphotericin B
	Cisplatin and carboplatin
	Zoledronic acid Iodinated contrast
	Fleet sodium phosphate
	Snake venom
	**Endogenous**
	Myoglobin
	Haemoglobin Bile salts
	Uric acid
	Phosphate
	Light chains

**Table 2:** Common nephrotoxins affecting the glomeruli.

Glomerular Disease	Nephrotoxin
Focal and segmental glomerulosclerosis	Zoledronic acid
	Interferon
	Heroin
	Pamidronate
	Doxorubicin
	Calcineurin inhibitors, e.g., cyclosporin
	Anabolic steroids
Membranous nephropathy	Non-steroidal anti-inflammatory drugs
	Penicillamine
	Tumour necrosis factor-α inhibitor
	Immune checkpoint inhibitors, e.g., pembrolizumab
	Mercury
Minimal change disease	Pamidronate Antibiotics, e.g., penicillin, cephalosporin, rifampicin Non-steroidal anti-inflammatory drugs Lithium

**Table 3:** Common nephrotoxins affecting the renal microvasculature.

Microvascular Disease	Nephrotoxin
Thrombotic microangiopathy	Quinine
	Calcineurin inhibitors, e.g., cyclosporin
	Vascular endothelial growth factor inhibitor, e.g., bevacizumab
	Trimethoprim-sulfamethoxazole
	Gemcitabine
	Clopidogrel
	Cocaine

## Data Availability

No new data were created or analyzed in this study.
